# The impact of folate and vitamin B12 status on cognitive function and brain atrophy in healthy elderly and demented Austrians, a retrospective cohort study

**DOI:** 10.18632/aging.103714

**Published:** 2020-07-24

**Authors:** Jasmin Rabensteiner, Edith Hofer, Günter Fauler, Eva Fritz-Petrin, Thomas Benke, Peter Dal-Bianco, Gerhard Ransmayr, Reinhold Schmidt, Markus Herrmann

**Affiliations:** 1 Clinical Institute of Medical and Chemical Laboratory Diagnostics, Medical University Graz, Graz, Austria; 2 Clinical Division of Neurogeriatrics, Department of Neurology, Medical University of Graz, Austria; 3 Institute for Medical Informatics, Statistics and Documentation, Medical University of Graz, Austria; 4 Department of Neurology, Medical University of Innsbruck, Innsbruck, Austria; 5 Department of Neurology, Medical University of Vienna, Vienna, Austria; 6 Department of Neurology 2, Med Campus III, Faculty of Medicine, Johannes Kepler University, Kepler University Hospital, Linz, Austria

**Keywords:** vitamin B12, folate, homocysteine, dementia, cognitive function

## Abstract

Background: Dementia, and in particular Alzheimer’s disease (AD), is a debilitating progressive disease with high prevalence in our society. Vitamin B12 and folate deficiency are potential modifiable risk factors. However, previous studies reported inconsistent results.

Results: The average concentrations of all biochemical markers were within the respective reference ranges. Cross-sectional and longitudinal analyses did not reveal significant associations between biochemical markers and cognitive function, global or regional brain volume, cortical thickness or cortical surface area, neither in controls nor in AD patients.

Conclusions: Variations of direct and indirect markers of B12 and folate status are not associated with cognitive dysfunction and brain atrophy.

Methods: This retrospective study explored the association between biochemical markers of B12 and folate status, cognitive function and MRI-based brain atrophy in cognitive normal elderly (controls) and AD patients. Folate, total and active vitamin B12 and MMA were measured in blood samples from 378 controls and 217 AD patients. Neuropsychiatric tests capturing memory, executive function and visuopractical skills were performed in all participants. Brain atrophy was assessed by MRI in 155 controls and 217 AD patients. In a subset of participants cognitive testing (n=234) and MRI (n=182) was repeated after an average median between 1.25 and 6.25 years.

## INTRODUCTION

Dementia, and in particular Alzheimer’s disease (AD), is a debilitating progressive disease with high prevalence in our ever aging societies that puts enormous burden on patients, their families and public health care systems.

At present, curative therapies are lacking and preventive measures that potentially delay onset and progression of the disease are of particular importance. Factors known to lower the incidence of Alzheimer´s disease are educational attainment, social integration, regular physical activity, treatment of vascular risk factors at midlife. Nutrition is another potentially disease modifying factor [1]. Vitamin B12 (B12) and folate are essential factors in pathways that have repeatedly been linked to neurodegeneration [2], such as one-carbon metabolism, DNA-methylation and nucleotide synthesis [3], and have also been related to cognitive decline and dementia but studies are so far inconclusive [4]. Both vitamins are required for efficient elimination of homocysteine (HCY), the cytotoxic product of the methionine cycle [3]. A lack of betain-homocysteine-methyltransferase (BHMT) in the central nervous system hampers the detoxification of HCY when intracellular B12 and folate levels are deficient [5, 6]. Furthermore, the limited supply of methyl-groups and nucleotides in B-vitamin deficient subjects impairs cell regeneration and proliferation.

In a systematic review of prospective cohort studies O'Leary F et al. did not find a clear link between serum B12 concentrations and cognitive decline [6]. Also, elevated plasma HCY, a functional marker of intracellular folate and B12 deficiency, increased the risk of dementia and cognitive decline in some studies [2, 7, 8], but not in others [9]. The inconsistent results of existing studies may, at least partly, be explained by methodological differences between studies including cohort composition and size, study duration, variable adjustment for confounders, and the choice of biochemical markers and cognitive outcome measure.

Few studies on the association of B-vitamins and cognitive functioning included structural brain imaging to assess whether the relationship, if any, is mediated by global or regional neurodegenerative changes or vascular lesions including small vessel disease related abnormalities such as lacunar stroke or white matter lesions. Vogiatzoglou A et al. demonstrated that low B12, but not low folate or high HCY, is associated with accelerated brain volume loss in elderly individuals without dementia [10]. Furthermore, B-vitamin supplementation appears to slow down brain atrophy [11], but has no effect on cognition itself [12, 13]. Additionally, white matter lesions have been shown to be an early phenomenon of blood-brain barrier dysfunction in the development of small vessel disease and an increase in periventricular white matter lesions in patients with lacunar stroke has been shown to be related to lower B12 status [14].

The present study aimed to address the inconsistent results described above by measuring direct and indirect biochemical markers of B12 and folate status in a well-characterized prospective cohort of demented patients and community-dwelling non-demented elderly Austrians. Biochemical results were used to explore cross-sectional and longitudinal associations with cognitive function, MRI-based brain atrophy and vascular abnormalities.

## RESULTS

**Figure 1:**
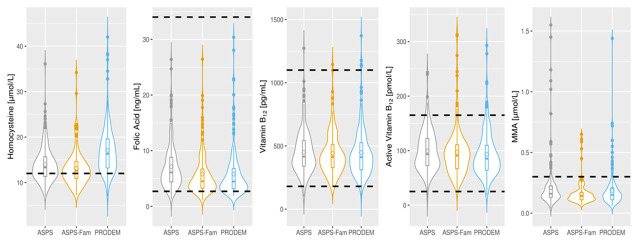
**Distribution of vitamin B laboratory parameters in normal elderly individuals (ASPS N=223, ASPS-Fam N=155) and in AD patients (PRODEM N=217).** The circle within the boxplot denotes the mean of the parameter. Dotted lines represent the reference range or clinical cut-off of each parameter. Homocysteine: < 12 μmol/L; Folate: 2.7-34 ng/mL; Vitamin B_12_: 180-1100 pg/mL; Active Vitamin B_12_: 25-165 pmol/L; MMA: <0.3 μmol/L. AD: Alzheimer’s disease; MMA: methyl malonic acid, ASPS: Austrian Stroke Prevention Study; ASPS-Fam: Austrian Stroke Prevention Family Study; PRODEM: Prospective Dementia Registry.

Demographics, frequency of risk factors and laboratory findings are displayed in Table 1 and Figure 1. All three cohorts included more females then males, and the controls are younger than the AD patients. HCY concentrations are mainly above the upper risk limit of 12 μmol/L in all three cohorts, but values in control subjects are lower than in AD patients. Folate levels are mainly located at the lower end of the reference range with higher levels in ASPS than in ASPS-Fam and PRODEM. Total B12, active B12 and MMA values fall well within the corresponding reference range.

As can be seen from Table 1 and Figure 1, Median HCY concentrations were within the respective reference ranges for males and females in all three cohorts, but values of the ASPS (normal elderly) were lower than in PRODEM (AD patients). However, the majority of participants exhibited HCY concentrations above 12 μmol/L, which is associated with an increased vascular risk.

**Table 1:** Demographics, risk factors and Vitamin B laboratory parameters.

**Cohort**	**Reference Range**	**ASPS**	**ASPS-Fam**	**PRODEM**	p*
N		223	155	217	
females, N (%)		141 (63.2%)	100 (64.5%)	126 (58.1%)	0.38
age (years), median [IQR]		69.00 [64.00 - 75.00]	67.00 [56.00 - 72.00]	76.00 [70.00 - 80.00]	3.34E-24
education (years), median [IQR]		10 [9-10]	10 [10 - 13]	10 [9-13]	5.50E-7
hypertension, N (%)		168 (75.3%)	96 (61.9%)	144 (66.4%)	0.02
diabetes, N (%)		27 (12.1%)	15 (9.7%)	34 (15.7%)	0.22
atrial fibrillation, N (%)		11 (4.9%)	9 (5.8%)	18 (8.3%)	0.33
eGFR, median [IQR]		63.43 [57.03 – 69.10]	74.17 [64.57 – 85.10]	NA**	5.01E-17
homocysteine [μmol/L], median [IQR]	4.4-13.6 (females) 5.5-16.2 (males)	13.30 [11.30-15.70]	12.60 [10.90 - 14.60]	16.30 [13.20 - 19.65]	2.17E-19
folate [ng/mL], median [IQR]	2.7 – 34	6.11 [4.36-8.86]	4.51 [3.26 - 6.73]	4.46 [3.14 - 6.82]	7.59E-8
vitamin B_12_ [pg/mL], median [IQR]	180-1100	415.30 [337.20-544.20]	412.10 [327.90 - 512.10]	411.60 [310.75 - 525.80]	0.69
active vitamin B_12_ [pmol/L], median [IQR]	25-165	93.37 [72.31-122.80]	90.67 [66.68 - 110.80]	85.10 [64.14 - 110.35]	0.04
MMA [μmol/L], median [IQR]	<0.3	0.16 [0.13-0.23]	0.14 [0.11 - 0.17]	0.15 [0.11 - 0.21]	0.001

*p-value of the comparison. Chi-Square test was used to compare categorical variables and Kruskal-Wallis-Test was used to compare the non-normally distributed continuous variables between ASPS, ASPS-Fam and PRODEM. The Mann-Whitney-U Test was used to compare the eGFR between ASPS and ASPS-Fam.

** eGFR is not available (NA) in PRODEM.

ASPS: Austrian Stroke Prevention Study; ASPS-Fam: Austrian Stroke Prevention Family Study; PRODEM: Prospective Dementia Registry; eGFR: estimated glomerular filtration rate; MMA: methyl malonic acid.

### Vitamin B-status and cognition

As shown in Table 2, in both, normal elderly and AD patients, associations between Vitamin B laboratory findings and cognitive function were not significant after correction the number of all statistical tests in the table. Similar results were obtained for the comparison of cognitive function between individuals in the highest versus the lowest concentration of each biochemical analyte (Table 3). Also, normal elderly individuals and AD patients with the highest probability for a functional vitamin deficiency (those with high HCY and low folate or B12 concentrations) performed similar on cognitive testing than those with the lowest probability for functional vitamin deficiency (low HCY and high folate or B12 concentration) (Table 4).

**Table 2:** Association between Vitamin B laboratory parameters and cognition in normal elderly (ASPS + ASPS-Fam.) and AD patients (PRODEM).

	**Cognition**	**N**	**Homocysteine**			**Folate**			**Vitamin B_12_**			**Active Vitamin B_12_**			**MMA**		
			**β**	**SE**	**p~**	**β**	**SE**	**p~**	**β**	**SE**	**p~**	**β**	**SE**	**p~**	**β**	**SE**	**p~**
Normal elderly *																	
(ASPS+ ASPS-Fam)	memory	378	-3.20E-03	1.08E-02	0.973	-9.35E-04	9.73E-03	0.973	1.46E-04	2.27E-04	0.895	1.14E-03	9.50E-04	0.814	-2.04E-03	2.49E-01	0.993
	executive function	378	-5.95E-03	6.83E-03	0.814	-6.67E-03	6.08E-03	0.814	9.64E-06	1.43E-04	0.973	1.53E-04	5.96E-04	0.973	-2.74E-01	1.59E-01	0.531
	visuopractical skills	378	-2.08E-02	1.04E-02	0.433^§^	2.59E-02	9.39E-03	0.300^§^	-1.36E-04	2.21E-04	0.895	1.27E-04	9.28E-04	0.973	1.58E-01	2.43E-01	0.895
	general cognition	378	-5.51E-03	1.03E-02	0.958	-1.21E-03	9.31E-03	0.973	4.57E-05	2.18E-04	0.973	1.54E-04	9.12E-04	0.973	-1.65E-01	2.40E-01	0.895
	memory - annualized change	93	1.11E-02	4.81E-03	0.325^§^	2.22E-03	3.35E-03	0.895	-4.99E-06	8.72E-05	0.973	-7.97E-05	3.12E-04	0.973	-1.93E-01	1.10E-01	0.531
	executive function - annualized change	93	-4.98E-03	3.88E-03	0.814	5.19E-03	2.67E-03	0.433	-1.44E-05	6.71E-05	0.973	2.79E-04	2.42E-04	0.814	3.01E-02	8.55E-02	0.973
	visuopractical skills - annualized change	93	-8.93E-03	6.26E-03	0.700	4.06E-03	4.38E-03	0.814	-1.11E-04	1.08E-04	0.814	-4.40E-05	3.78E-04	0.973	3.05E-01	1.37E-01	0.325^§^
	general cognition - annualized change	93	2.23E-03	5.04E-03	0.969	2.91E-03	3.51E-03	0.814	3.58E-05	8.70E-05	0.971	-1.12E-04	3.13E-04	0.973	-1.70E-01	1.10E-01	0.605
AD patients**																	
(PRODEM)	CERAD sum score	217	-2.27E-01	9.38E-02	0.325^§^	1.34E-01	1.13E-01	0.814	2.38E-03	2.60E-03	0.814	1.25E-02	1.21E-02	0.814	-3.15E+00	3.77E+00	0.814
	CERAD sum score - annualized change	141	-1.87E-01	1.12E-01	0.550	1.32E-01	1.36E-01	0.814	1.41E-03	3.06E-03	0.969	-6.11E-03	1.31E-02	0.969	-3.64E+00	3.99E+00	0.814

*Normal elderly individuals: the cognitive domains memory, executive function, visuopractical skills and general cognition were assessed in the pooled ASPS and ASPS-Fam data.

**Dementia patients: The sum score of the CERAD test battery was assessed in PRODEM participants.

All analyses are adjusted for age, sex, hypertension, diabetes, atrial fibrillation and education.

Homocysteine analyses in normal elderly individuals are additionally adjusted for eGFR (calculated using the CKDEpi formula); eGFR was not available in dementia patients.

The analyses in the normal elderly were additionally adjusted for the cohort, as data of ASPS and ASPS-Fam were combined in these analyses.

~p-values are false discovery rate adjusted for multiple testing.

^§^p-value < 0.05 before false discovery rate correction for multiple testing

AD: Alzheimer’s disease, MMA: methyl malonic acid, ASPS: Austrian Stroke Prevention Study, ASPS-Fam: Austrian Stroke Prevention Family Study, PRODEM: Prospective Dementia Registry; N: number of individuals in analyses, β: regression coefficient, SE: standard error of regression coefficient, p: p-value

**Table 3:** Comparison of baseline cognition between the lowest and the highest quartile of Vitamin B laboratory parameters in normal elderly (ASPS + ASPS-Fam.) and AD patients (PRODEM).

	**Cognition**	**Homocysteine**				**Folate**				**Vitamin B_12_**				**Active Vitamin B_12_**				**MMA**			
		**N**	**β**	**SE**	**p~**	**N**	**β**	**SE**	**p~**	**N**	**β**	**SE**	**p~**	**N**	**β**	**SE**	**p~**	**N**	**β**	**SE**	**p~**
Normal elderly* (ASPS + ASPS-Fam)	memory	194	0.159	0.13	0.82	190	0.046	0.13	0.92	190	0.131	0.11	0.82	188	0.108	0.12	0.82	202	0.120	0.11	0.82
	executive function	194	-0.048	0.08	0.92	190	-0.084	0.08	0.82	190	0.021	0.07	0.92	188	-0.037	0.07	0.92	202	-0.068	0.07	0.82
	visuopractical skills	194	-0.228	0.12	0.82	190	0.195	0.11	0.82	190	-0.023	0.10	0.92	188	0.043	0.12	0.92	202	0.032	0.11	0.92
	general cognition	194	0.023	0.12	0.92	190	0.001	0.12	1.00	190	0.061	0.10	0.92	188	-0.001	0.12	1.00	202	-0.026	0.11	0.92
AD patients** (PRODEM)	CERAD sum score	110	-1.006	1.35	0.92	110	1.806	1.38	0.82	109	1.498	1.50	0.82	109	1.000	1.45	0.92	108	2.180	1.40	0.82

*Normal elderly individuals: the cognitive domains memory, executive function, visuopractical skills and general cognition were assessed in the pooled ASPS and ASPS-Fam data.

** Dementia patients: The sum score of the CERAD test battery was assessed in PRODEM participants.

All analyses are adjusted for age, sex, hypertension, diabetes, atrial fibrillation and education. Homocysteine analyses in normal elderly individuals are additionally adjusted for eGFR (calculated using the CKDEpi formula), eGFR was not available in dementia patients.

The analyses in the normal elderly were additionally adjusted for the cohort, as data of ASPS and ASPS-Fam were combined in these analyses.

~ The p-values are false discovery rate adjusted for multiple testing. AD: Alzheimer’s disease, MMA: methyl malonic acid, ASPS: Austrian Stroke Prevention Study, ASPS-Fam: Austrian Stroke Prevention Family Study, PRODEM: Prospective Dementia Registry; N: number of individuals in analyses, β: regression coefficient, SE: standard error of regression coefficient, p: p-value

**Table 4:** Comparison of baseline cognition between individuals with the highest versus the lowest probability of functional vitamin deficiency in normal elderly individuals and in AD patients.

	**Cognition**	**Q1 Vitamin B_12_ & Q4 Homocysteine~**			**Q1 Folate & Q4 Homocysteine^#^**		
		**β**	**SE**	**P^#^**	**β**	**SE**	**P^#^**
Normal elderly * (ASPS+ ASPS-Fam)	memory	-0.11	0.13	0.70	-0.02	0.13	0.96
	executive function	-0.09	0.08	0.70	0.07	0.08	0.70
	visuopractical skills	-0.10	0.12	0.70	-0.07	0.12	0.84
	general cognition	-0.12	0.12	0.70	0.02	0.12	0.96
AD patients**	CERAD sum score	-0.08	1.57	0.96	-1.82	1.52	0.70

~Individuals with the highest probability of functional vitamin deficiency (homocysteine levels in the highest quartile and vitamin B_12_ levels in the lowest quartile, N_normal elderly_=40, N_AD_=25) compared to individuals with the lowest probability of functional vitamin deficiency (homocysteine levels in the 3 lowest quartiles and vitamin B_12_ levels in the 3 highest quartiles, N_normal elderly_=338, N_AD_=192). ^#^Individuals with the highest probability of functional vitamin deficiency (homocysteine levels in the highest quartile and folate levels in the lowest quartile, N_normal elderly_=40, N_AD_=26) compared to individuals with the lowest probability of functional vitamin deficiency (homocysteine levels in the 3 lowest quartiles and folate levels in the 3 highest quartiles, N_normal elderly_=338, N_AD_=191). *Normal elderly individuals: the cognitive domains memory, executive function, visuopractical skills and general cognition were assessed in the pooled ASPS and ASPS-Fam data. ** Dementia patients: The sum score of the CERAD test battery was assessed in PRODEM participants. All analyses are adjusted for age, sex, hypertension, diabetes, atrial fibrillation and education. Homocysteine analyses in normal elderly individuals are additionally adjusted for eGFR (calculated using the CKDEpi formula), eGFR was not available in AD patients. The analyses in the normal elderly were additionally adjusted for the cohort, as data of ASPS and ASPS-Fam were combined in these analyses. ^#^ The p-values are false discovery rate adjusted for multiple testing. AD: Alzheimer’s disease, ASPS: Austrian Stroke Prevention Study, ASPS-Fam: Austrian Stroke Prevention Family Study, PRODEM: Prospective Dementia Registry. β:regression coefficient, SE: standard error of regression coefficient, p:p-value.

Similarly to the cross-sectional analyses, longitudinal analyses did not reveal significant associations between biochemical markers of B-vitamin status and cognition after correction for multiple testing (Table 2).

### Vitamin B-status and MRI

None of the biochemical markers of B-vitamin status related to global or regional brain volume, cortical thickness or cortical surface area, neither in normal elderly individuals nor in AD patients (Supplementary Table 1). Similar results were obtained for the comparison of MRI variables in individuals with the highest and the lowest concentration of each biochemical analyte (Supplementary Table 2) and for those with the highest versus the lowest probability of functional vitamin deficiency (Table 5). There were also no significant associations between vitamin B status and annualized change of MRI markers (Supplementary Table 3).

**Table 5:** Comparison of baseline MRI between individuals with the highest versus the lowest probability of functional vitamin deficiency in normal elderly individuals and in AD patients.

	**Q1 VitB12 & Q4 Homocysteine**						**Q1 Folate & Q4 Homocysteine**					
	**Normal elderly**			**AD patients**			**Normal elderly**			**AD patients**		
	**β**	**SE**	**P^#^**	**β**	**SE**	**P^#^**	**β**	**SE**	**P^#^**	**β**	**SE**	**P^#^**
Total Gray Matter Volume	6.95E-03	4.84E-03	0.768	-3.62E-03	6.54E-03	0.934	7.26E-03	4.96E-03	0.768	-2.51E-03	6.35E-03	0.996
Subcortical Gray Matter Volume	8.62E-04	5.71E-04	0.768	-1.24E-03	6.67E-04	0.683	1.21E-03	5.83E-04	0.563^§^	-6.84E-04	6.51E-04	0.768
Hippocampal Volume	1.55E-05	6.56E-05	0.996	-2.19E-04	8.52E-05	0.235^§^	6.78E-05	6.78E-05	0.768	-1.35E-05	8.39E-05	0.996
**Cortical Volume**												
Total	2.87E-03	3.86E-03	0.900	-1.94E-04	5.75E-03	0.996	3.92E-03	3.95E-03	0.768	-9.28E-04	5.57E-03	0.996
Frontal Lobe	1.77E-03	1.63E-03	0.768	-1.20E-05	2.20E-03	0.996	2.10E-03	1.67E-03	0.768	-7.16E-05	2.14E-03	0.996
Temporal Lobe	1.13E-03	9.10E-04	0.768	-4.52E-04	1.44E-03	0.996	-5.60E-05	9.37E-04	0.996	-9.74E-04	1.39E-03	0.900
Parietal Lobe	-4.16E-05	1.11E-03	0.996	-3.67E-04	1.44E-03	0.996	6.24E-04	1.14E-03	0.934	-5.03E-04	1.39E-03	0.996
Occipital Lobe	3.36E-04	5.50E-04	0.934	1.23E-04	5.99E-04	0.996	9.10E-04	5.58E-04	0.768	7.17E-04	5.79E-04	0.768
**Cortical Thickness**												
Frontal Lobe	-1.66E-04	3.27E-04	0.954	4.07E-04	4.49E-04	0.810	4.70E-04	3.33E-04	0.768	-1.07E-05	4.36E-04	0.996
Temporal Lobe	-2.18E-04	3.04E-04	0.900	4.89E-05	5.18E-04	0.996	3.07E-04	3.11E-04	0.768	-3.46E-04	5.02E-04	0.990
Parietal Lobe	-3.36E-04	2.92E-04	0.768	7.23E-05	3.86E-04	0.996	4.08E-04	2.99E-04	0.768	-1.82E-04	3.74E-04	0.955
Occipital Lobe	-3.10E-05	2.26E-04	0.996	2.67E-04	2.56E-04	0.768	3.17E-04	2.30E-04	0.768	1.14E-04	2.49E-04	0.963
**Cortical Surface Area**												
Frontal Lobe	1.58E-01	6.04E-02	0.235^§^	-7.95E-02	7.58E-02	0.768	-1.81E-02	6.35E-02	0.996	6.83E-03	7.38E-02	0.996
Temporal Lobe	9.08E-02	2.96E-02	0.128^§^	-2.82E-02	4.27E-02	0.907	-4.82E-02	3.12E-02	0.768	3.59E-03	4.14E-02	0.996
Parietal Lobe	8.87E-02	4.41E-02	0.563^§^	-2.93E-02	5.36E-02	0.934	-3.61E-02	4.58E-02	0.888	1.58E-02	5.20E-02	0.996
Occipital Lobe	2.78E-02	2.74E-02	0.768	-2.43E-02	2.93E-02	0.868	2.53E-02	2.80E-02	0.810	3.56E-02	2.84E-02	0.768

~Individuals with the highest probability of functional vitamin deficiency (homocysteine levels in the highest quartile and vitamin B_12_ levels in the lowest quartile, N_normal elderly_=20, N_AD_=25) compared to individuals with the lowest probability of functional vitamin deficiency (homocysteine levels in the 3 lowest quartiles and vitamin B_12_ levels in the 3 highest quartiles, N_normal elderly_=135, N_AD_=192). ^#^Individuals with the highest probability of functional vitamin deficiency (homocysteine levels in the highest quartile and folate levels in the lowest quartile, N_normal elderly_=19, N_AD_=26) compared to individuals with the lowest probability of functional vitamin deficiency (homocysteine levels in the 3 lowest quartiles and folate levels in the 3 highest quartiles, N_normal elderly_=136, N_AD_=191). Normal elderly individuals from ASPS-Fam (N=155), AD patients from PRODEM (N=217). All analyses are adjusted for age, sex, hypertension, diabetes and atrial fibrillation. Homocysteine analyses in normal elderly individuals are additionally adjusted for eGFR (calculated using CKDEpi formula), eGFR was not available in AD patients. AD: Alzheimer’s disease, ASPS: Austrian Stroke Prevention Study, ASPS-Fam: Austrian Stroke Prevention Family Study, PRODEM: Prospective Dementia Registry. β: regression coefficient, SE: standard error of regression coefficient, p:p-value. ^#^ p-values are false discovery rate adjusted for multiple testing. ^§^ p-value < 0.05 before false discovery rate correction for multiple testing.

## DISCUSSION

The present results show that neither direct nor functional markers of folate and B12 status are associated with cognitive function and brain atrophy in controls and AD patients that are well supplied with these two vitamins. In contrast to most previous studies, we here combined a comprehensive panel of direct and functional biochemical markers with a detailed assessment of cognitive function and morphological features of brain atrophy and vascular lesions. In addition, the prospective study design allowed for longitudinal assessment, which confirmed the observations of the cross-sectional analyses.

Although the primary statistical analyses of the present data showed some significant associations, these associations disappeared after multiple testing correction which was performed to reduce the number of false positive findings that occur by chance when many statistical tests are carried out. We therefore consider these few significant associations to be chance findings. Even when focusing on individuals in the lowest quartiles of the individual vitamin concentrations and those with the highest probability of functional B-vitamin deficiency (combining the highest tHCY concentration [Q4] with the lowest vitamin concentration [Q1]) results did not change. Considering that average B12 and folate concentrations of ASPS, ASPS-Fam and PRODEM participants were well within the respective reference ranges, it can be concluded that differences of these vitamins within the respective reference ranges are unlikely to modify the risk of dementia and brain volume loss. However, the present results do not exclude relevant effects in B-vitamin deficient individuals. To explore this hypothesis we compared cognitive function and brain atrophy in individuals with tHCY concentrations >15 μmol/L and <12 μmol/L. This comparison revealed reduced visual practical skills and a lower cortical surface area in hyperhomocysteinemic individuals. However, after adjustment for multiple testing these differences lost significance.

Previous observational studies that explored the role of folate and B12 for cognitive function and brain atrophy yielded heterogeneous results [15–20]. For example, in the OHAP study, holoTC, MMA and tHcy predicted cognitive decline [15]. Subjects with a holoTC concentration of 100 pmol/L had a 30% slower rate of cognitive decline than those with 50 pmol/L. Folate instead was unrelated to cognitive function. Also the prospective Chicago Health and Aging Project revealed significant associations between MMA, tHCY, cognitive function and brain atrophy [20]. Vogiatzoglou et al. performed yearly MRI and blood biomarker studies over a 5 year period in 107 community-dwelling elderly individuals. The odds ratio for accelerated brain loss was approximately 6 times higher amongst subjects in the lowest tertile of B12 (<308 pmol/L) and active B12 (<54 pmol/L) [10]. Interestingly, high levels of MMA or tHCY and low levels of folate were not associated with brain volume loss in this study. [21, 22]. In the Swedish Study on Aging and Care in Kungsholmen (SNAC-K) with 2570 dementia free participants neither direct indices of B12 nor red blood cell folate were related to incident dementia and structural brain changes over a period of 6 years [18]. However, tHCY was significantly associated with an increased risk of incident dementia, and the methionine to tHCY ratio predicted brain atrophy. A later subgroup analysis of participants with repeated MRI scans showed a longitudinal relationship between B12, holo-TC and brain loss. In contrast to the studies discussed before, the Hordaland Health Study with 2203 dementia free elderly Norwegians (aged 72-74 years) showed a correlation between plasma folate and cognitive performance [16]. Similarly, Ravaglia et al. demonstrated an association between low folate concentrations and an increased risk for AD in dementia-free Italians during a follow-up period of 4 years [*2*].

The heterogenous results of observational studies are at least partly due to differences in the study cohorts. Major factors that may have affected the results are age, prevalence of vitamin deficiencies at baseline and vitamin supplementation. Despite the fact that none of the study participants of ASPS, ASPS-Fam and PRODEM used vitamin supplements, the majority of participants were well within the respective reference range of folate, B12, active B12 and MMA suggesting an adequate B-vitamin status in the Austrian population. The rather low percentage of B-vitamin deficient subjects limits the number of individuals at risk for potential adverse effects. Additional aspects to consider are follow-up time and different methods to assess cognitive function.

Further insights can be obtained from B-vitamin supplementation studies, such as the folate and FACIT trial [23] and the VITACOG study [11]. In the FACIT trial three years of oral folate supplementation reduced HCY by 26% and improved several domains of cognitive function including memory, information processing and sensorimotor speed [23]. In the VITACOG study supplementation of 271 elderly with high doses of folate, vitamin B6 and B12 for 24 months resulted in a 24% reduction of brain atrophy. In addition, a greater rate of brain atrophy was associated with lower final cognitive test scores. Another 2-year RCT with 400 μg of folate and 100 μg of B12 revealed a significant improvement in cognitive function [24]. However, this study assessed cognitive function by telephone interviews.

The present study has a number of strengths and weaknesses. In contrast to most other studies, we analysed a significant number of dementia patients alongside healthy elderly individuals, which widens the scope of the results. The comprehensive panel of direct and indirect markers of B12 and folate status allows the detection of early stages of B12 and folate deficiency [21, 22]. Another strength is the use of MRI to assess atrophic changes in the total brain and regions that are known to be affected by AD. Evaluation of cognitive functioning was thorough and captured various domains of cognition. The main weaknesses of the present study are the relatively young age of the participants and the rather good supply with B12 and folate. This implies that the inherent risk of dementia in control subjects was rather low. Furthermore, vitamin B6, another co-factor involved in the degradation of HCY, was not measured. The sample size of our study was considerable but lower than in other studies [15, 16]. Sample size is a particular issue in the longitudinal analyses of the current investigation, which lack statistical power. Another weakness of our study is the differential follow-up between risk groups and the relatively short follow-up time, particularly in demented patients. The longest follow-up period in our samples was 6 years, and the likelihood is high that this is too short for the detection of significant longitudinal effects, especially when the follow-up cohort is rather small and well supplied with B12 and folate. However, the negative results for cognitive function are supported by MRI results, which also show no association between B-vitamin status and brain atrophy. Additionally, the low number of individuals with vascular lesions in the MRI data prevented a meaningful statistical analysis.

In conclusion, elderly Austrians are well supplied with B12 and folate. Variations of direct and indirect markers of B12 and folate status are not associated with cognitive dysfunction and brain atrophy. Therefore, supplementation of B12 and folate in subjects with adequate direct and indirect biochemical indices of these two vitamins is unlikely to exert beneficial short to mid-term preventive effects on cognitive dysfunction and brain parenchymal loss. The present results do not exclude such effects of B12 and folate supplementation in individuals with vitamin deficiency.

## MATERIALS AND METHODS

### Study design

Stored blood samples from 378 normal elderly individuals (controls) and 217 AD patients were used to measure direct and indirect markers of B12 and folate status. Cognitive function of all participants was analysed by neuropsychiatric tests capturing memory, executive function and visuopractical skills. In 155 controls and 217 AD patients MRI of the brain was performed in order to capture neurodegenerative and vascular changes. The present study included only subjects with a complete set of biochemical and cognitive test results who did not take any folate or vitamin B supplements. Results were used to explore potential associations between B-vitamin status, cognitive function and structural markers of neurodegeneration.

In a subset of subjects, data from follow-up examinations were available and the annualized change was calculated for cognition in 93 controls and 141 AD patients and longitudinal changes of MRI measures were assessed in 41 controls and 141 AD patients.

### Study cohorts

The study population was composed of the following three cohorts: Austrian Stroke Prevention Study (ASPS), Austrian Stroke Prevention Family Study (ASPS-Fam), and Prospective Registry on Dementia in Austria (PRODEM). The ASPS was established in 1991 as a prospective single-centre study to investigate the effects of vascular risk factors on brain structure and function [25, 26]. Participants were randomly selected from the community register of Graz (Austria), and were included if they were free of stroke and dementia and had a normal neurologic examination. Between 2006 and 2013, participants of ASPS and their first-grade relatives were invited to join ASPS-Fam, an extension of ASPS with a similar study protocol [27, 28].

**Figure 2:**
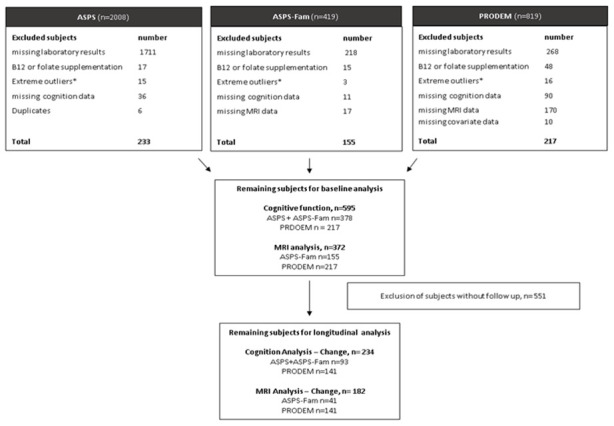
**Selection of study samples.** ASPS: Austrian Stroke Prevention Study, ASPS-Fam: Austrian Stroke Prevention Family Study, PRODEM: Prospective Dementia Registry. *Extreme outliners: mean+3x standard deviation.

PRODEM is a longitudinal multi-center cohort study on dementia [29]. It started in 2009 and included individuals with a dementia diagnosis according to the DSM-IV criteria. The diagnosis of dementia patients in the current study was either probable (N=137) or possible (N=80) AD. The selection of participants for the final study cohort is shown in Figure 2. All study protocols were approved by the ethics committee of the Medical University of Graz, Austria, and written informed consent was obtained from all participants.

### Vitamin B status

HCY, folate, total B12 and active B12 (Holotranscobalamin II) were measured in stored serum and plasma samples using commercial electrochemiluminescence immunoassays (ECLIA) from Roche Diagnostics that were run on a COBAS 8000 e 602 analyser (Roche Diagnostics, Switzerland).

Serum methyl-malonic acid (MMA) was determined by an in-house high performance liquid chromatography (HPLC) mass spectrometry (MS) method using deuterated MMA (d3-MMA) as internal standard. Pre-analytical sample preparation included liquid/liquid extraction (ethyl acetate) of 300 μl of serum followed by derivatization to butyl esters with butanol/HCl. Subsequently, samples were separated on a HPLC system (Thermo Scientific) equipped with a C-18 column (3.3cm x 4.6mm, 3μ, Supelco 58977) and a methanol/water gradient. Finally, high resolution-mass spectrometry analysis was conducted using Orbitrap-technology (QExactive, Thermo Scientific) in positive ionization and full scan mode. Quantitation of MMA was performed by direct determination of peak area ratios of MMA (m/z = 231.1596, retention time = 8.04 min) and d3-MMA (m/z = 234.1785, retention time = 8.03 min).

### Neuropsychological testing

In both groups, cognitive function was assessed with dedicated test batteries tailored to assess cognition in healthy adults and in AD patients. A detailed description of these test batteries has been published previously [26, 30–35]. In order to reduce sources of measurement error, we used composite measures of cognitive function in the analyses rather than the results of individual tests. These summary measures were calculated by converting test results to z-scores based on the mean and standard deviation of the combined ASPS and ASPS-Fam sample, and by computing the average z-scores within each cognitive domain. For individuals with follow-up examinations, we calculated the follow-up z-scores based on mean and standard deviation of the baseline to be able to directly compare the baseline and follow-up. As the follow-up time varied between studies (ASPS median [IQR]: 3.17 years [3.00-3.33], ASPS-Fam: 6.25 [5.50 - 6.75], PRODEM: 1.25 [1-2]), we calculated an annual change. The annual change in cognitive domains was defined as the difference between baseline and follow-up z-scores divided by the follow-up time.

Additionally, principal components analysis was used to calculate a measure of global cognitive ability combining the results from all individual tests. The unrotated first component defined the general cognitive ability factor (g-factor) [36]. The annual change in the g-factor was defined the difference between baseline and follow-up g-factor divided by the follow-up time.

In the PRODEM study, cognitive function was assessed with the “Consortium to Establish a Registry for Alzheimer’s disease“(CERAD-Plus) test battery [37] which was specifically developed for dementia patients. The CERAD-Plus test is made up of several subtests assessing verbal fluency, verbal and non-verbal memory, visuo-constructional abilities, executive function and word finding. The scores of the CERAD-Plus subtests were transformed into z-scores corrected for age, sex, and education based on normative data and these z-scores were added up to create a sum score of the CERAD-Plus test battery. The annual change in the CERAD sum score was defined as the difference between baseline and follow-up CERAD sum score divided by the follow-up time.

### Magnetic resonance imaging (MRI)

ASPS-Fam participants underwent MRI on a 3T whole-body MR system (TimTrio; Siemens Healthcare, Erlangen, Germany). PRODEM MRI scans were obtained on a 3T scanner (Magnetom TrioTim; Siemens Healthcare, Erlangen, Germany) and on several 1,5 T scanners (Magnetom TimTrio, Magnetom Avanto and Symphony TIM; Siemens Healthcare, Erlangen, Germany). All centers used a standardized MRI protocol including a high-resolution T1- weighted 3D MPRAGE sequence covering the whole brain and an axial T2-weighted-FLAIR sequence. MRI scans from ASPS participants could not be considered in the present study as they obtained in the 1990s, when 3D T1 and FLAIR sequences were not yet available.

Total, cortical and subcortical gray matter volume, hippocampus volume and lobar cortical volume, thickness and surface area were computed from the T1 weighted MPRAGE images using FreeSurfer 5.3 [38, 39]. Based on the intensity of the voxels in the MRI image the software automatically segments the brain into subcortical gray volumetric structures, such as the hippocampus, and cortical gray matter. Freesurfer divides the cerebral cortex into gyral based regions of interest and provides the cortical volume, surface area and thickness for each of these regions. Values of these regions were added up or averaged for volume and surface area, and cortical thickness, respectively, to obtain these measures for the lobes. To correct for variations in individual head size, all measures were normalized for total intracranial volume. For individuals with follow-up examinations, the annual change is the ratio between the follow-up and the baseline value divided by the follow-up time.

### Statistical analysis

Statistical analysis was performed using the ‘R’ version 3.6.1. Violin plots showing the distribution of the laboratory parameters were generated. Normality of quantitative variables was assessed by visual inspection of the qq-plot and Shapiro-Wilk’s test. Normally distributed variables are reported as mean ± standard deviation (std) and non-normally distributed variables as median and interquartile range (IQR). Demographics, risk factors and laboratory parameters were compared between ASPS, ASPS-Fam and PRODEM using Chi-Square test for categorical variables and Kruskal-Wallis-Test or Mann-Whitney-U test for non-normally distributed continuous variables. We determined the association between each laboratory parameter and baseline cognition as well as baseline MRI in normal elderly individuals and in AD patients separately using three different models. The first model included the laboratory parameter as a continuous predictor. The second model compared cognition and MRI between the highest (Q4) and the lowest (Q1) quartile of the laboratory parameter by including a binary predictor coding the quartile status. In the third model, where the outcome measures were obtained from the MRI studies, subjects were compared according to quartiles of HCY, B12, and folate concentrations. The MRI measures in the highest quartile of each of the vitamins was compared to the MRI measures in the lowest quartile of each of the vitamins. The association between each laboratory parameter and change in cognition as well as change in MRI was determined using a model including the change as the outcome and the laboratory parameter as a continuous predictor. In AD patients, associations were tested using multiple linear regression analysis. In the controls, we used mixed models with the family structure as a random effect to account for the relatedness in ASPS-Fam. A kinship matrix describing the degree of relationship between any two individuals in the study was also generated. All analyses were adjusted for age, sex, hypertension, diabetes and atrial fibrillation. Cognition analyses were additionally adjusted for education, and HCY analyses in normal elderly were additionally adjusted for eGFR. For cognition analyses in control subjects, we pooled ASPS and ASPS-Fam data. To adjust for any undetected differences between ASPS and ASPS-Fam, we used the study as a covariate in cognition analyses. As the risk of false positive associations increases with the number of hypotheses tests, we applied false discovery rate (FDR) correction [40] to all p-values within each table, except for Table 1. The FDR method compensates for the number of tests by adjusting the p-values to control for the proportion of false positives. Violin plots showing the distribution of the laboratory parameters were generated using the R packages ggplot2 [41] and cowplot [42].

## Supplementary Material

Supplementary Table 1

Supplementary Table 2

Supplementary Table 3
