# Supplementary material for: The impact of folate and vitamin B12 status on cognitive function and brain atrophy in healthy elderly and demented Austrians, a retrospective cohort study
Source: Aging (Albany NY). 2020 Jul 24;12(15):15478–91. doi: 10.18632/aging.103714 (PMC7467363; doi:10.18632/aging.103714)
Supplement: Supplementary Table 1 [file aging-12-103714-s003..docx]

**Supplementary Table 1. Association between Vitamin B laboratory parameters and baseline MRI in normal elderly individuals (N=155) and AD patients (N=217). (To be continued)**

|  | **Homocysteine** | | | | | | **Folate** | | | | | | **Vitamin B_12_** | | | | | |
| --- | --- | --- | --- | --- | --- | --- | --- | --- | --- | --- | --- | --- | --- | --- | --- | --- | --- | --- |
|  | **Normal elderly** | | | **AD patients** | | | **Normal elderly** | | | **AD patients** | | | **Normal elderly** | | | **AD patients** | | |
|  | β | **SE** | **p~** | β | **SE** | **p~** | β | **SE** | **p~** | β | **SE** | **p~** | β | **SE** | **p~** | β | **SE** | **p~** |
| Total Gray Matter Volume | 5.52E-04 | 4.68E-04 | 0.996 | -2.11E-04 | 3.97E-04 | 0.996 | 1.70E-04 | 3.95E-04 | 0.996 | -2.40E-05 | 4.75E-04 | 0.996 | 3.45E-07 | 9.51E-06 | 0.996 | 1.43E-05 | 1.08E-05 | 0.996 |
| Subcortical Gray Matter Volume | 1.08E-04 | 5.48E-05 | 0.996^§^ | -4.37E-05 | 4.07E-05 | 0.996 | -3.54E-05 | 4.68E-05 | 0.996 | -8.86E-05 | 4.85E-05 | 0.996 | -4.84E-07 | 1.12E-06 | 0.996 | 2.53E-07 | 1.11E-06 | 0.996 |
| Hippocampus Volume | 6.12E-06 | 6.24E-06 | 0.996 | -6.06E-06 | 5.23E-06 | 0.996 | -3.83E-06 | 5.48E-06 | 0.996 | -8.27E-06 | 6.26E-06 | 0.996 | -8.77E-08 | 1.27E-07 | 0.996 | 1.22E-07 | 1.43E-07 | 0.996 |
| **Cortical Volume** |  |  |  |  |  |  |  |  |  |  |  |  |  |  |  |  |  |  |
| Total | 2.32E-04 | 3.72E-04 | 0.996 | -9.65E-05 | 3.48E-04 | 0.996 | 2.11E-04 | 3.15E-04 | 0.996 | 6.81E-06 | 4.17E-04 | 0.996 | 2.21E-06 | 7.54E-06 | 0.996 | 1.27E-05 | 9.44E-06 | 0.996 |
| Frontal Lobe | 8.31E-05 | 1.57E-04 | 0.996 | -4.73E-05 | 1.34E-04 | 0.996 | 6.05E-05 | 1.34E-04 | 0.996 | 1.04E-05 | 1.60E-04 | 0.996 | -6.12E-07 | 3.19E-06 | 0.996 | 4.87E-06 | 3.62E-06 | 0.996 |
| Temporal Lobe | 3.93E-05 | 8.83E-05 | 0.996 | -2.69E-05 | 8.72E-05 | 0.996 | 1.25E-04 | 7.26E-05 | 0.996 | -5.23E-05 | 1.04E-04 | 0.996 | 7.35E-07 | 1.78E-06 | 0.996 | 3.21E-06 | 2.36E-06 | 0.996 |
| Parietal Lobe | 3.65E-05 | 1.07E-04 | 0.996 | -7.72E-06 | 8.72E-05 | 0.996 | 5.36E-05 | 9.04E-05 | 0.996 | 1.00E-04 | 1.04E-04 | 0.996 | 1.06E-06 | 2.17E-06 | 0.996 | 2.35E-06 | 2.37E-06 | 0.996 |
| Occipital Lobe | 4.95E-05 | 5.30E-05 | 0.996 | 4.32E-05 | 3.62E-05 | 0.996 | -2.06E-05 | 4.46E-05 | 0.996 | -2.10E-05 | 4.35E-05 | 0.996 | 8.26E-07 | 1.07E-06 | 0.996 | -1.15E-07 | 9.89E-07 | 0.996 |
| **Cortical Thickness** |  |  |  |  |  |  |  |  |  |  |  |  |  |  |  |  |  |  |
| Frontal Lobe | 2.57E-05 | 3.17E-05 | 0.996 | -1.32E-05 | 2.73E-05 | 0.996 | -1.29E-06 | 2.58E-05 | 0.996 | -6.07E-06 | 3.27E-05 | 0.996 | -2.99E-07 | 6.35E-07 | 0.996 | 1.26E-06 | 7.38E-07 | 0.996 |
| Temporal Lobe | 1.44E-05 | 2.94E-05 | 0.996 | -2.34E-05 | 3.14E-05 | 0.996 | -1.85E-07 | 2.47E-05 | 0.996 | -3.29E-05 | 3.76E-05 | 0.996 | 1.81E-07 | 5.94E-07 | 0.996 | 1.49E-06 | 8.50E-07 | 0.996 |
| Parietal Lobe | 1.12E-05 | 2.85E-05 | 0.996 | -1.92E-05 | 2.34E-05 | 0.996 | 1.31E-06 | 2.33E-05 | 0.996 | 1.74E-05 | 2.80E-05 | 0.996 | 4.10E-10 | 5.72E-07 | 1.000 | 1.19E-06 | 6.32E-07 | 0.996 |
| Occipital Lobe | -3.71E-07 | 2.18E-05 | 0.996 | 5.96E-06 | 1.56E-05 | 0.996 | 1.46E-05 | 1.82E-05 | 0.996 | -1.43E-05 | 1.86E-05 | 0.996 | 7.97E-08 | 4.41E-07 | 0.996 | 3.05E-07 | 4.23E-07 | 0.996 |
| **Cortical Surface Area** |  |  |  |  |  |  |  |  |  |  |  |  |  |  |  |  |  |  |
| Frontal Lobe | -1.24E-03 | 6.03E-03 | 0.996 | 1.63E-03 | 4.61E-03 | 0.996 | 3.20E-03 | 4.91E-03 | 0.996 | -1.11E-04 | 5.52E-03 | 0.996 | 2.91E-05 | 1.21E-04 | 0.996 | -1.88E-04 | 1.25E-04 | 0.996 |
| Temporal Lobe | -7.20E-04 | 2.98E-03 | 0.996 | 2.40E-03 | 2.59E-03 | 0.996 | 4.85E-03 | 2.41E-03 | 0.996^§^ | 9.97E-04 | 3.10E-03 | 0.996 | 1.61E-05 | 6.01E-05 | 0.996 | -8.10E-05 | 7.03E-05 | 0.996 |
| Parietal Lobe | 4.22E-04 | 4.34E-03 | 0.996 | 3.60E-03 | 3.24E-03 | 0.996 | 2.09E-03 | 3.58E-03 | 0.996 | 9.08E-04 | 3.89E-03 | 0.996 | 4.44E-05 | 8.74E-05 | 0.996 | -1.42E-04 | 8.80E-05 | 0.996 |
| Occipital Lobe | 3.12E-03 | 2.65E-03 | 0.996 | 1.54E-03 | 1.78E-03 | 0.996 | -2.77E-03 | 2.14E-03 | 0.996 | 5.33E-04 | 2.13E-03 | 0.996 | 4.54E-05 | 5.33E-05 | 0.996 | -2.83E-05 | 4.84E-05 | 0.996 |

Normal elderly individuals from ASPS-Fam (N=155), AD patients from PRODEM (N=217).
All analyses are adjusted for age, sex, hypertension, diabetes and atrial fibrillation. Homocysteine analyses in normal elderly individuals are additionally adjusted for eGFR (calculated using CKDEpi formula), eGFR was not available in dementia patients.
All Freesurfer Variables are normalised for total intracranial volume
The p-values are false discovery rate adjusted for multiple testing.
^§^ p-value < 0.05 before false discovery rate correction for multiple testing.
AD: Alzheimer’s disease, MMA: methyl malonic acid, ASPS-Fam: Austrian Stroke Prevention Family Study, PRODEM: Prospective Dementia Registry; N: number of individuals in analyses, β: regression coefficient, SE: standard error of regression coefficient, p:p-value.

**Supplementary Table 1 Association between Vitamin B laboratory parameters and baseline MRI in normal elderly individuals (N=155) and AD patients (N=217). (Continued)**

|  | **Active Vitamin B_12_** | | | | | | **MMA** | | | | | |
| --- | --- | --- | --- | --- | --- | --- | --- | --- | --- | --- | --- | --- |
|  | **Normal elderly** | | | **AD patients** | | | **Normal elderly** | | | **AD patients** | | |
|  | β | **SE** | **p~** | β | **SE** | **p~** | β | **SE** | **p~** | β | **SE** | **p~** |
| Total Gray Matter Volume | 1.21E-05 | 3.42E-05 | 0.996 | 2.60E-05 | 5.08E-05 | 0.996 | -6.57E-03 | 1.82E-02 | 0.996 | 3.16E-04 | 1.58E-02 | 0.996 |
| Subcortical Gray Matter Volume | -4.31E-07 | 4.04E-06 | 0.996 | -1.25E-06 | 5.22E-06 | 0.996 | -1.51E-03 | 2.14E-03 | 0.996 | -7.13E-04 | 1.62E-03 | 0.996 |
| Hippocampus Volume | 1.20E-07 | 4.65E-07 | 0.996 | 3.59E-07 | 6.71E-07 | 0.996 | -4.74E-05 | 2.41E-04 | 0.996 | -3.74E-04 | 2.07E-04 | 0.996 |
| **Cortical Volume** |  |  |  |  |  |  |  |  |  |  |  |  |
| Total | 1.97E-05 | 2.71E-05 | 0.996 | 1.56E-05 | 4.46E-05 | 0.996 | 1.21E-03 | 1.44E-02 | 0.996 | 6.39E-03 | 1.38E-02 | 0.996 |
| Frontal Lobe | 3.45E-07 | 1.15E-05 | 0.996 | 3.96E-07 | 1.71E-05 | 0.996 | 4.02E-03 | 6.05E-03 | 0.996 | 3.54E-03 | 5.31E-03 | 0.996 |
| Temporal Lobe | 7.72E-06 | 6.37E-06 | 0.996 | 1.22E-05 | 1.11E-05 | 0.996 | -1.44E-03 | 3.42E-03 | 0.996 | 1.47E-03 | 3.46E-03 | 0.996 |
| Parietal Lobe | 5.30E-06 | 7.80E-06 | 0.996 | 5.76E-06 | 1.12E-05 | 0.996 | -1.86E-03 | 4.13E-03 | 0.996 | -5.67E-05 | 3.47E-03 | 0.996 |
| Occipital Lobe | 5.67E-06 | 3.85E-06 | 0.996 | -1.72E-07 | 4.65E-06 | 0.996 | 8.07E-04 | 2.05E-03 | 0.996 | 3.42E-04 | 1.44E-03 | 0.996 |
| **Cortical Thickness** |  |  |  |  |  |  |  |  |  |  |  |  |
| Frontal Lobe | -4.18E-07 | 2.27E-06 | 0.996 | 4.81E-07 | 3.49E-06 | 0.996 | -9.50E-04 | 1.24E-03 | 0.996 | 5.79E-04 | 1.08E-03 | 0.996 |
| Temporal Lobe | 2.57E-06 | 2.13E-06 | 0.996 | 3.20E-06 | 4.02E-06 | 0.996 | -6.94E-04 | 1.14E-03 | 0.996 | 1.81E-04 | 1.25E-03 | 0.996 |
| Parietal Lobe | 3.71E-07 | 2.04E-06 | 0.996 | 2.38E-06 | 2.99E-06 | 0.996 | 3.37E-04 | 1.11E-03 | 0.996 | -1.76E-04 | 9.31E-04 | 0.996 |
| Occipital Lobe | 1.22E-06 | 1.58E-06 | 0.996 | -4.37E-07 | 1.99E-06 | 0.996 | 4.66E-04 | 8.42E-04 | 0.996 | 2.67E-04 | 6.19E-04 | 0.996 |
| **Cortical Surface Area** |  |  |  |  |  |  |  |  |  |  |  |  |
| Frontal Lobe | 3.06E-05 | 4.31E-04 | 0.996 | -6.10E-04 | 5.89E-04 | 0.996 | 4.00E-01 | 2.33E-01 | 0.996 | 2.13E-02 | 1.83E-01 | 0.996 |
| Temporal Lobe | -2.08E-05 | 2.15E-04 | 0.996 | 7.95E-05 | 3.32E-04 | 0.996 | -1.04E-02 | 1.17E-01 | 0.996 | 9.36E-03 | 1.03E-01 | 0.996 |
| Parietal Lobe | 1.40E-04 | 3.13E-04 | 0.996 | -3.26E-04 | 4.16E-04 | 0.996 | -6.16E-02 | 1.70E-01 | 0.996 | -3.69E-02 | 1.29E-01 | 0.996 |
| Occipital Lobe | 2.05E-04 | 1.92E-04 | 0.996 | 4.92E-05 | 2.28E-04 | 0.996 | 3.83E-02 | 1.04E-01 | 0.996 | -2.87E-02 | 7.07E-02 | 0.996 |

Normal elderly individuals from ASPS-Fam (N=155), AD patients from PRODEM (N=217).
All analyses are adjusted for age, sex, hypertension, diabetes and atrial fibrillation. Homocysteine analyses in normal elderly individuals are additionally adjusted for eGFR (calculated using CKDEpi formula), eGFR was not available in dementia patients.
All Freesurfer Variables are normalised for total intracranial volume
The p-values are false discovery rate adjusted for multiple testing. p-value < 0.05 before false discovery rate correction for multiple testing.
AD: Alzheimer’s disease, MMA: methyl malonic acid, ASPS-Fam: Austrian Stroke Prevention Family Study, PRODEM: Prospective Dementia Registry;N: number of individuals in analyses, β: regression coefficient, SE: standard error of regression coefficient, p:p-value.
